# How are the cellular functions of myosin VI regulated within the cell?

**DOI:** 10.1016/j.bbrc.2007.11.150

**Published:** 2008-04-25

**Authors:** Folma Buss, John Kendrick-Jones

**Affiliations:** a Cambridge Institute for Medical Research, University of Cambridge, Wellcome Trust/MRC Building, Hills Road, Cambridge CB2 2XY, UK; b MRC Laboratory of Molecular Biology, Hills Road, Cambridge CB2 2QH, UK

**Keywords:** Motor proteins, Myosin VI, Actin, Cytoskeleton, PtdIns(4,5)P_2_, Membrane trafficking, Myosin phosphorylation, Optineurin, Dab2, GIPC

## Abstract

This review, dedicated to the memory of Professor Setsuro Ebashi, focuses on our current work investigating the cellular functions and regulation of the unique unconventional motor, myosin VI. This myosin, unlike all the other myosins so far studied, moves towards the minus end of actin filaments and has been implicated in a wide range of cellular processes such as endocytosis, exocytosis, cell migration, cell division and cytokinesis. Myosin VI’s involvement in these cellular pathways is mediated by its interaction with specific adaptor proteins and is regulated by multiple regulatory signals and modifications such as calcium ions, PtdIns(4,5)P_2_ (PIP_2_) and phosphorylation. Understanding the functions of myosin VI within the cell and how it is regulated is now of utmost importance given the recent observations that it is associated with a number of human disorders such as deafness and cancers.

Our review is dedicated to the memory of Professor Setsuro Ebashi and his pioneering work on the fundamental role of calcium ions as a cellular signal and regulator of intracellular processes, which has had such a profound influence on our understanding of the regulation of muscular contraction. We would like to start by briefly introducing what we believe are a few of the major milestones in the discovery of the mechanisms involved in regulating contractile activity in cells. We will focus mainly on myosin linked regulatory systems first in muscle and then in cells in general focusing on our current work investigating how the functions of an unconventional myosin (myosin VI) may be regulated in non-muscle cells. Our selected milestones are the following:

(1) Almost forty years ago in 1968 Professors Setsuro Ebashi and Makoto Endo published their famous review on “Calcium Ion and Muscle Contraction” [1]. It described their fundamental studies over at least the previous 10 years demonstrating that calcium ions were the regulatory signal controlling muscular contraction. It also described the discovery by Ebashi and his colleagues of the calcium regulatory complex, troponin, on the actin filaments in vertebrate skeletal muscle. This work was a major breakthrough in our understanding of how contraction is regulated by Ca^2+^ in vertebrate skeletal and cardiac muscles. Further elegant studies by Ebashi [2] and a number of other groups including those of Perry, Gergely, and Hartshorne [3–5] established that the troponin complex was composed of three components, troponin-C (TnC), the subunit which binds the Ca^2+^, troponin-I (TnI), the subunit that inhibits actin-myosin interaction and troponin-T (TnT) the tropomyosin binding subunit which binds the complex to the actin filaments. Furthermore they demonstrated that Ca^2+^ binding to TnC overcomes the inhibitory effect of TnI and thus acting as an ON/OFF switch activates muscular contraction.
(2) The discovery in 1970 of the Ca^2+^ regulatory mechanism in molluscan myosins [6] was a complete surprise since up until then it was believed that Ca^2+^ regulation of muscular contraction was mediated solely by the troponin complex on the actin thin filaments. However in molluscan muscles it was shown that Ca^2+^ regulation of contraction was mediated by Ca^2+^ binding directly to the myosin and that the regulatory light chains located in the ‘lever-arm’ or neck region of the myosin were the regulatory subunits which under Ca^2+^ control regulated myosin interaction with actin and thus contraction in these muscles [6,7]. It was later demonstrated that all myosins contain regulatory light chains although those in vertebrate striated and cardiac muscles do not serve as calcium regulatory subunits [8].
(3) In the mid 1970s another Ca^2+^ regulatory mechanism was identified in vertebrate smooth muscles and non-muscle cells [9]. In these cells Ca^2+^ regulates actin-myosin interaction indirectly by binding to calmodulin, which activates a myosin light chain kinase (MLCK) to phosphorylate the regulatory light chains (RLCs) on the myosin. RLC phosphorylation switches on the myosin so that it can interact with actin to generate force and movement [10–12]. In non-muscle cells RLC phosphorylation may also control the assembly of the myosin molecules into filaments that are able to move and generate force when interacting with actin [13]. At least *in vitro* when the RLCs are non-phosphorylated these myosins are monomeric and in a compact “switched off” folded state with their tails bent up next to their motor domains and when their RLCs are phosphorylated these myosin tails unfold and assemble into filaments that are able to interact with actin filaments to generate force and movement [13]. Although we have not yet succeeded in demonstrating that this regulated myosin filament assembly process occurs *in vivo* it is believed that such a mechanism is crucial for many cellular processes such as assembling and disassembling the contractile ring required for cytokinesis.
(4) In the mid 1990s it was shown that myosin existed as a large superfamily of motor proteins [14–17] with multiple functions and a possibly diverse range of regulatory mechanisms. So far 24 distinct classes of myosins have been identified [18]. In humans, 40 myosins belonging to 12 separate classes are expressed [17]. Each class of myosin has a basic N-terminal motor domain, RLCs or calmodulin subunits bound in the “neck” or lever-arm region and tail domains that differ widely in their size and structure. Unlike the myosins in muscle that form filaments and assemble into relatively stable sarcomeric structures optimised for muscular contraction, the ‘new’ myosins do not assemble into filaments but are organised in a variety of flexible actin cytoskeletal-membrane assembles optimised for their diverse cellular functions [19].

In this review we have focused on the cellular functions and possible regulatory mechanisms of one of these ‘new’ myosins, myosin VI. It is the only class of myosin that moves towards the minus end of actin filaments [20] and therefore appears to have unique functions in the cell. Given the believed polarity of actin filaments in the cell with their plus (barbed) ends inserted into or at membranes and their minus (pointed) ends projecting inwards then myosin VI would move cargo from the plasma membrane into the cell and away from the surface of organelles such as the Golgi complex. Myosin VI has been localised in membrane ruffles at the leading edge of the cell, at the Golgi complex, in clathrin coated pits/vesicles at the plasma membrane, at the centrosome and in the midbody during cytokinesis and has been implicated in a wide range of processes such as endocytosis, exocytosis, maintenance of Golgi morphology, cytokinesis and cell migration [21]. To begin to understand the role(s) that myosin VI plays in these cellular events we will first examine its structure and *in vitro* properties.

## Domain structure and *in vitro* properties of myosin VI

Myosin VI is composed of the following basic myosin domains: an N-terminal canonical 80 kDa motor domain with an ATP binding pocket and actin binding interface, a short neck or ‘lever-arm’ region with a single IQ domain that binds a calmodulin, a tail with helical regions and a C-terminal cargo binding domain [22]. Myosin VI also contains a number of unique inserts: in the motor domain next to the switch 1 loop beside the ATP binding pocket there is a 22 amino acid insert that controls the nucleotide on and off rates and is thus thought to fine tune myosin VI for its specific cellular functions [23] and between the motor domain and ‘lever-arm’ region there is a 53 amino acid insert that is the reverse gear responsible for the retrograde movement of myosin VI along actin filaments [20]. Within the tail region there are a large and small insert that generate four alternatively spliced myosin VI isoforms that are differentially expressed and have distinct intracellular locations and functions [24,25].

High resolution structural analysis of the myosin VI motor domain and lever-arm in the post-power stroke state reveals a structure that is very similar to that of the motor domains of the myosin IIs and Vs with the exception of the two unique inserts [26]. The 53 amino acid insert is absent in all other myosin classes and contains a novel motif that binds a non-exchangeable calmodulin subunit [27]. This insert is an integral part of the converter region that redirects the lever-arm of myosin VI by 120^o^ towards the minus end of the actin filament thus allowing myosin VI to move in the reverse direction along these filaments [26]. So far the structure of none of the myosin VI tail domains have been solved.

**Fig. 1:**
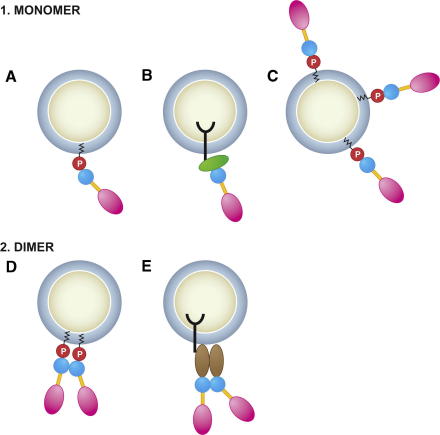
Myosin VI could function as a monomer or dimer or as both in the cell. Shown are a few possible ways how myosin VI (motor domain in red, tail region in yellow and cargo-binding domain (CBD) in blue) may associate with vesicles and move them around the cell. As a monomer (A) it could bind via its CBD to PIP_2_ (red P) inserted in the membrane; or in (B) it could bind to a binding partner such as Dab2 (shown in green) that is bound to the cytoplasmic tail of a membrane receptor; or (C) multiple monomers could bind via PIP_2_ molecules to the vesicle in clusters or distributed uniformly all over the vesicle. As a dimer (D) the CBDs of two myosin VIs could dimerise when bound to PIP_2_ molecules clustered on the vesicle surface or in (E) when two myosin VIs bind to a dimeric binding partner (shown in brown) that is bound to a membrane receptor on a vesicle.

Kinetic studies have shown that myosin VI has a high affinity for ADP and ADP release is the rate limiting step in its ATPase cycle [28]. Thus myosin VI is a high duty ratio motor protein and with a duty ratio of 0.8 it spends most (∼80%) of its steady state ATPase cycle strongly bound to actin [28]. Since it was believed that myosin VI existed as a dimeric molecule on the basis of a predicted coiled coil region in its tail region, most recent kinetic studies have used myosin VI constructs that have been dimerised by the inclusion of a leucine zipper instead of the C-terminal cargo binding tail domain [28–30] These engineered artificial dimers, containing two motor domains, have a high duty ratio and move processively along actin filaments with an unexpectedly large step size of 30–36 nm [31,32]. Like myosin V the two motor domains in these myosin VI dimers appear to communicate with each other (gating between the heads) so as to move processively along an actin filament in a hand over hand fashion [33,34]. A number of possible models have been proposed to explain how gating might operate in these myosin VI dimers [35]. However the observation that full-length expressed and native myosin VI containing the crucial C-terminal cargo binding region are monomers by biochemical and biophysical criteria and move non-processively along actin filaments with single steps of 18 nm [36] questions the relevance of the work on the artificial dimers and raises the intriguing proposal that myosin VI may exist and function as a non-processive monomer and possibly as a processive dimer in the cell? (Fig. 1).

**Fig. 2:**
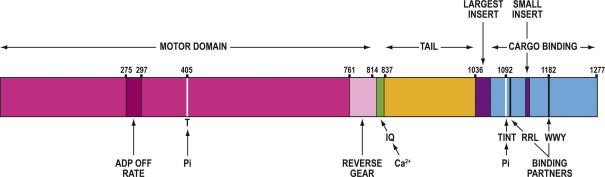
A cartoon illustrating the positions of the potential regulatory sites in myosin VI. In the motor domain there is a 22 amino acid insert (275–297) which regulates the ADP off rate and thus ATP binding to the ATPase site; a threonine at 405 in the actin binding interface which maybe phosphorylated and involved in modulating interaction with actin and in the converter region, a unique 53 aa insert (761–814) that is the reverse gear, which determines the direction that the myosin moves along an actin filament. The neck region contains a single IQ motif (in green) that binds a calmodulin, the regulatory subunit that binds calcium. Just before the cargo-binding domain (CBD) there is a large insert (1036–1060) and within the CBD a small insert (1140–1148) that generate four alternatively spliced isoforms that are differentially expressed and have distinct intracellular locations and functions. In the CBD, there are two ‘hot spots’, the RRL and WWY motifs, where all the so far characterised myosin VI binding partners bind (Table 1); also there are two threonines (1092 and 1094) in the TINT sequence that can be phosphorylated and regulate the binding of optineurin to the CBD.

The long step size achieved by myosin VI (30–36 nm for the artificial dimer and 18 nm for the monomer) was unexpected since it has such a short lever-arm (maximum predicted length 10 nm)? It obviously cannot generate such a large step using the conventional lever-arm mechanism proposed for myosins II and V where the step size depends in a linear fashion on the length of the lever-arm [37–39]. Although a working model has been proposed to explain how a myosin VI dimer might be able to achieve such a large step size [31], whether it exists as a stable dimer *in vivo*, the overall conformation of the full-length molecule especially the structure and stability of the helical tail and how it is regulated are some of the properties that first need to be determined. Thus it is apparent that the ‘new’ classes of unconventional myosins are extremely versatile multi-domain motors in which inserts or domains can be added, modified or exchanged to create ‘novel’ motor proteins with unique properties capable of travelling towards the minus or plus ends of actin filaments [40] (see Fig. 2).

## Intracellular functions of myosin VI in mammalian cells

To illustrate the functional diversity of myosin VI, we will focus on just three of its cellular functions; those which have been studied in greatest detail:

### Myosin VI and clathrin-mediated endocytosis

At the plasma membrane, endocytosis is the complex process by which a cell is able to communicate with its extracellular environment and involves the regulated uptake of proteins, nutrients, receptors, and the retrieval of membrane proteins and lipids lost after exocytosis. Although clathrin mediated endocytosis is the best characterised pathway, at least three other uptake pathways have now been identified: macropinocytosis, caveolae-mediated endocytosis and clathrin- and caveolae-independent endocytosis [41,42]. Clathrin mediated endocytosis involves the following basic sequence of steps: (i) cargo binds to its receptor and clusters on the plasma membrane; (ii) a pit forms containing these cargo-receptor complexes and clathrin starts to assembles around it; (iii) the plasma membrane invaginates to form a clathrin coated vesicle; (iv) membrane fission occurs and the clathrin coated vesicle is released into the cell where (v) the clathrin coat is removed and the vesicle is transported towards its destination in the cell. Each step requires a vast assembly of structural coat components and a host of accessory proteins that includes actin cytoskeletal and motor proteins, which cooperate together to drive the endocytic machinery [42–44].

So which specific steps in this complex process involves myosin VI? The answer to this question appears to vary depending on the type of cell being studied and myosin VI isoform expressed [25,45]. For example in polarised epithelial cells, the myosin VI isoform with the large insert in the tail domain is concentrated in clathrin coated pits and vesicles in the terminal web region just below the apical plasma membrane [46,47]. At this apical surface Disabled 2 (Dab2), a myosin VI binding partner, co-localises with myosin VI and since Dab2 binds to the cytoplasmic tails of members of the LDLR family it links myosin VI to these cell surface receptors [48]. So at the start of endocytosis, myosin VI is first recruited to the Dab2/LDL receptor complex at the plasma membrane by binding to Dab2 and the signalling molecule PIP_2_ [49]. Given the polarity of the actin filaments with their plus ends at the plasma membrane and their minus ends pointing into the cell, myosin VI being a minus end directed motor could move the PIP_2_/Dab2/receptor complexes along the membrane and cluster them to start to form a clathrin-coated pit. Myosin VI could then generate the force required for vesicle formation by moving along these actin filaments and by pulling in the membrane together with the host of structural coat and accessory proteins, a number of which sense membrane curvature, could form a clathrin-coated pit/vesicle. By pushing polymerising actin filaments into the vesicle neck in conjunction with accessory proteins like dynamin, myosin VI could lead to vesicle scission and could subsequently transport the clathrin-coated vesicle into the actin rich terminal web region where it is uncoated. In the final stages the myosin VI isoforms with the small insert or with no inserts in the tail may move the vesicles through the cortical actin filament network before they fuse with the early endosome compartment [45]. Considerable further work is needed to verify these speculations and establish if and how myosin VI works in these specific steps in endocytosis.

#### Myosin VI and exocytosis at the Golgi complex

The Golgi complex plays a central role in the protein biosynthesis pathway in cells. Every protein synthesised in the ER is transported to the Golgi complex, where it undergoes post-translational modification and is sorted for delivery to either the cell surface or to an intracellular compartment. In mammalian cells the Golgi complex occupies a central perinuclear position organised around the microtubule organising centre (MTOC) and consists of stacks of flattened membrane interconnected cisternae which can be classified as the *cis*, *medial*, and *trans*-Golgi compartments [50]. The *cis*- and *trans*-sides of the Golgi complex are surrounded by an extensive network of membrane tubules and vesicles; the *cis*-Golgi network (CGN) and the *trans*-Golgi network (TGN). Proteins from the ER enter the Golgi complex at the CGN, are passed through the complex and are sorted and packaged into a variety of different vesicular carriers at the TGN for dispatch to different cellular locations [51].

Myosin VI is present in vesicles at the Golgi complex that are on average 200–400 nm away from the *trans*-side of the Golgi complex [52,53]. In fibroblasts from the Snell’s waltzer myosin VI knock-out mouse the absence of myosin VI leads to a 40% reduction in the size and a collapse of the reticulate Golgi network structure and a 40% reduction in protein secretion from the TGN to the plasma membrane [53]. Both of these phenotypes can be rescued by transfection and expression of full-length myosin VI. So myosin VI interacting with the actin cytoskeleton surrounding the Golgi complex could be involved in maintaining the elaborate reticulate structure of the Golgi membranes. Whereas in the secretory pathway myosin VI could also be involved in: (1) sorting proteins into different subdomains at the TGN for delivery to different cellular compartments or (2) it could play a role in vesicle formation and budding in analogy to its proposed endocytic function at the plasma membrane or (3) the short-range transport of cargo vesicles from the TGN towards microtubules for their long distance transport to the cell surface.

Myosin VI targeting to the Golgi complex and its involvement in the secretory pathway requires binding to one of its binding partners, optineurin [54] and the small G-protein Rab8, which is involved in membrane trafficking and has been identified as an optineurin binding partner [55]. In polarised epithelial cells to maintain their highly polarised shape, specialised pathways have evolved to sort and transport newly synthesised membrane proteins from the Golgi complex to either the basolateral or the apical surface depending on the type of sorting motifs present in these proteins [56–58]. In polarised MDCK cells the myosin VI isoform with no insert in the tail domain is required for sorting and transport of newly synthesised basolateral membrane proteins containing the tyrosine-sorting motif to the basolateral domain [47]. Since in polarised epithelial cells Rab8 acts as a membrane anchor and regulates transport to the basolateral domain [59,60] a functional transport complex containing myosin VI, optineurin and Rab8 may operate, since all three proteins are present in recycling endosomes, which are the sorting station for basolateral transport in MDCK cells [61].

#### Myosin VI and cell migration

A moving cell is polarised with a leading edge and a trailing edge and the protrusive force for forward movement is generated at the leading edge by the formation of pseudopodia or lamellipoda coupled with cell adhesion [62,63]. Identifying the role of myosin VI in cell migration has acquired greater urgency with the discovery that myosin VI is involved in cancer cell invasion [64]. So far the best example for myosin VI’s role in cell motility is in border cell migration in *Drosophila* ovaries, which has served as a model for cancer cell invasion [65,66]. Myosin VI is crucial for border cell migration and since it exists in a complex with the adhesion proteins E-cadherin and β-catenin it could by binding to stationary adhesion complexes in the membrane develop a protrusive force by pushing the actin filaments outwards thus moving the cell forwards [65]. In mammalian cells less is known about myosin VI’s role in cell motility although when stimulated with growth factors, myosin VI is actively recruited into membrane ruffles at the leading edge of these moving cells and is phosphorylated in the motor domain [52]. It has been suggested that in these cells F-actin polymerisation coupled with membrane insertion and cell adhesion generates the protrusive force at the front end of the cell to push the plasma membrane outwards [62]. Thus at the leading edge of the cell it is likely that the following processes drive cell migration: (1) polymerisation/depolymerisation of the actin filament network regulated by the Arp2/3 complex and the Rho GTPases [67,68] followed by the subsequent remodelling of the cortical actin cytoskeleton; (2) the assembly of adhesion protein complexes at the membrane and their adhesion to the substrate [69]; (3) the transport of new membrane and its insertion into the leading edge [52]; and (4) the development of protrusive force by motor proteins such as the myosins I, V, and VI interacting with the actin filaments and the stationary adhesion complexes in the membrane. In addition myosin VI together possibly with other myosins such as myosins Vs and Is are likely to be involved in the delivery of adhesion molecules and additional membrane to the leading edge of the moving cell.

So having briefly reviewed myosin VI’s possible roles in a number of basic cellular functions we will now discuss potential mechanisms how myosin VI’s involvement in these processes maybe regulated.

## Myosin VI regulation

Although relatively little is known about how myosin VI is regulated, multiple regulatory mechanisms are likely to be involved, acting in concert to modulate the activity of myosin VI rather than acting as an ON/OFF switch like the Ca^2+^ regulatory mechanisms that operate in muscle [21]. Since myosin VI is involved in such a wide variety of cellular functions it must be tightly regulated so that it is only active where and when it is required in the cell. For the cell to maintain its organisation and the correct distribution of components the activities of all the motor proteins involved must be tightly regulated and coordinated. Understanding how myosin VI is regulated has importance for developing therapeutic targets for combating human diseases such as cancer, deafness and cystic fibrosis. Possible myosin VI regulatory mechanisms include:

### Expression of alternatively spliced isoforms due to inserts in the tail domains

At the basic level of regulation myosin VI is expressed in a cell specific fashion as four alternatively spliced isoforms with a large (21–31 aa), or a small (9 aa), or both or no inserts in the tail region [70]. In polarised epithelial cells the myosin VI isoform with the large insert is localized at the apical surface where it is involved in clathrin-mediated endocytosis, whereas the myosin VI isoform with no inserts is localized to the *trans*-side of the Golgi complex and to recycling endosomes where it plays a role in the sorting and delivery of specific cargo to the basolateral surface [47]. How the presence or absence of these inserts in the tail influences the functions and intracellular targeting of myosin VI is not known. Since neither of the tail inserts contain any obvious structural motifs nor apparent binding sites they may control the overall conformation of the myosin VI molecule and thus possibly its binding to specific binding partners.

### Phosphorylation of potential sites in the motor domain and tail region

When A431 (a human epithelial carcinoma cell line) cells are stimulated with epidermal growth factor (EGF) myosin VI is recruited into the dynamic ruffling membrane at the leading edge and there is a fourfold increase in the level of myosin VI phosphorylation [52]. It was suggested that threonine (T_406_) in the hypotrophic cardiomyopathy loop in the actin binding region in the motor domain might be the residue phosphorylated and that PAK1, a Rac-, and cdc42-activated kinase, might be the kinase responsible. This T_406_ residue obeys the TEDS rule (it must be either a Thr, Glu, Asp or Ser) for myosin motor domain phosphorylation sites proposed by Bement and Mooseker [71]. Phosphorylation of this threonine residue in the motor domains of Acanthamoeba myosins IA and IB is required for maximal actin-activated ATPase activity [72], however in myosin VI phosphorylation of T_406_ does not alter its rate of actin filament sliding or its maximal actin-activated ATPase rate [29]. Although T_406_ phosphorylation has no apparent effect on myosin VI’s *in vitro* kinetic properties, in the cell it is possible that it may have a rather more subtle role modulating myosin VI-actin cytoskeletal interactions [52,73]. In the C-terminal region of the myosin VI tail two sites (T_1089_INT_1092_) phosphorylated by PAK3 have been identified by mass spectrometry and phosphorylation at these threonine residues has been shown to regulate optineurin binding to the myosin VI tail [54]. Interestingly phosphorylation at these sites does not regulate myosin VI binding to any of the other binding partners so far tested.

So far the kinases that have been tested on myosin VI are members of the PAK family, which exist as a number of isoforms with specific domains that target them to specific cellular locations. LMTK2 (Lemur tyrosine kinase2) was recently identified as a myosin VI binding partner, but so far it has not been demonstrated that it phosphorylates myosin VI [74]. It is possible that myosin VI acts as a transporter to carry LMTK2 to specific sites in the cell, where its phosphorylation target proteins are located. Thus trying to piece together the roles of myosin VI phosphorylation in both the motor and tail domains in a specific and temporal manner within the cell will be difficult but will be necessary to understand how these myosin VI regulatory systems operate.

### Ca^2+^ binding to the calmodulin subunit in the lever-arm region

Myosin VI has a short lever-arm with a single IQ motif that binds calmodulin but very little is known about how calcium binding to this calmodulin affects the structure and intracellular functions of the myosin. Although the unique insert (reverse gear) in the converter region, also contains a bound calmodulin, it appears that this calmodulin plays a structural role and its bound calcium is non-exchangeable [27]. Increases in Ca^2+^ concentration however do alter the *in vitro* kinetic properties of myosin VI; both the rate of ADP release and velocity of acto-myosin VI motility are reduced and in an artificial myosin VI dimer construct there is a significant decrease in processive movement [29]. Calcium also appears to be required for the binding of full-length myosin VI to liposomes [49] (see next section).

### Binding to phosphoinositides (PIP_2_) in the plasma membrane binding

Myosin VI binds to liposomes, specifically those containing the second messenger PtdIns(4,5)P_2_ (PIP_2_) [49]. The PIP_2_ binding site is in the C-terminal cargo binding tail region (CBD) and consists of a mix of basic residues (R/K) alternating with hydrophobic residues, very similar to the regions identified in other cytoskeletal PIP_2_-binding proteins [75]. How calcium binding to the calmodulin in the lever-arm controls PIP_2_ binding to the CBD is not known but it would suggest that major conformational changes in the myosin VI molecule may be involved (see next section). The myosin VI tail binds to PIP_2_ containing liposomes with high affinity (*K*_d_ = 0.3 μm) and when bound there is a major change (a 31% increase in α-helicity) in the secondary structure of the tail. In HeLa cells mutation of the PIP_2_ binding site reduces the targeting of full-length myosin VI and its tail to clathrin-coated structures by about 50% [49]. PIP_2_ is the major polyphosphoinositide in mammalian cells and acts as a second messenger and membrane anchor and is important for the attachment of the cytoskeleton to the plasma membrane, and for endocytosis, exocytosis and membrane trafficking. It recruits many cytoskeletal and endocytic proteins to the plasma membrane and regulates the assembly, scission and uncoating of clathrin-coated vesicles [76–78]. PIP_2_ is non-uniformly distributed in the plasma membrane and is concentrated at active sites of clathrin-mediated endocytosis where it may recruit the adaptor Dab2, myosin VI and accessory/cytoskeletal proteins to the membrane at the initiation of clathrin-coated pit assembly.

### Structural changes controlling the conformation of myosin VI

A number of proteins bind to the C-terminal domain in myosin VI (see “A monomer/dimer equilibrium” and Table 1) and a mechanism to regulate their binding is essential. Major conformational changes in the tail could be used to regulate/block these protein–protein interactions. For example non-muscle myosin IIs and myosin V can exist *in vitro* in a folded inactive state, with the tails interacting with the heads, regulated either by regulatory light chain phosphorylation or by calcium binding to calmodulin [13,79,80]. However whether these myosins exist in folded states in cells is yet to be established. So far there is no conclusive proof that myosin VI can exist as a folded molecule but its tail contains extensive regions which are predicted to be unstructured by multiple secondary structure prediction algorithms (performed using the NPS@.Network Protein Sequence Analysis web server) ([81] and there are helical regions with hinges that might also allow the tail to fold. Single molecule imaging studies show that myosin VI is a monomer and the tail is flexible; for example, in the absence of nucleotide, the tail is bent and lies next to the motor domain whereas in the presence of ATP the tail is straight [36]. Cellular localisation studies on myosin VI [52] support the idea that myosin VI may exist in an ‘inactive’ state in the cell since they show that a considerable proportion of the endogenous myosin VI is present as a diffuse cytoplasmic pool not associated with any obvious cellular compartments or structures. One envisages that in this cytoplasmic pool, myosin VI is in a folded inactive state with the binding sites on the CBD for its binding partners masked and the actin interface on the motor domain blocked and phosphorylation or increases in Ca^2+^ concentration and/or binding to cargo or membrane would activate the molecule.

### A monomer/dimer equilibrium

Myosin VI may function as both a non-processive monomer and a processive dimer depending on the cell’s requirements and controlling such a monomer–dimer equilibrium could be an important regulatory mechanism (see Fig. 1). One can imagine that for certain cellular functions such as maintaining tension, clustering transmembrane receptors or tethering vesicles to actin filaments, a non-processive monomer would operate whereas for retrograde transport of vesicles along actin filaments, processive dimers would be preferred. *In* *vitro* full-length myosin VI is a monomer however it can be induced to form a few dimers (17%) when clustered on actin filaments [82]. Many of myosin VI’s binding partners contain regions of predicted coiled coil and leucine zippers and there is preliminary evidence that one of them forms stable dimers [83]. Myosin VI binding to such a stable dimeric binding partner could then operate as a processive dimer. When the myosin VI C-terminal domain binds to liposomes it can be crosslinked to form dimers [49]. This is similar to the kinesin motor KIF1A/Unc104, which also dimerises upon liposome binding and the resulting dimer is able to transport the liposome processively along microtubules [84]. If myosin VI is involved in vesicular trafficking then it has been speculated that dimerisation on the surface of a vesicle or when clustered on actin filaments or when bound to specific binding partners could generate the most proficient transport motor [85,86] (Fig. 1). It is however very much a matter of debate whether dimer molecules are more efficient than multiple monomers in moving a 100nm vesicle through the highly cross-linked cortical actin network below the plasma membrane. However whether myosin VI exists and functions in the cell as a monomer and/or dimer and whether this process might act as a novel regulatory mechanism remains to be established.

### Binding to specific adaptors and binding partners

A number of binding partners that target/recruit myosin VI to specific intracellular locations and regulate/determine its functions have now been identified by yeast-2-hybrid screens, mammalian-2-hybrid assays and by a series of *in vitro* and *in vivo* binding assays (see Table 1). Four of them are briefly described here:

Disabled-2 (Dab2) is a 96-kDa protein, which is thought to be a specific adaptor for members of the Low Density Lipoprotein receptor (LDLR) family. Dab2 has a N-terminal phosphotyrosine domain that interacts not only with the internalisation signal in the cytoplasmic tail of LDLR receptors [87], but also simultaneously with phosphoinositides such as PIP2 thus attaching it to the plasma membrane [88]. Dab2 also binds to the adaptor protein AP2, to clathrin, to specific endocytic accessory proteins such as Eps 15 and to myosin VI [48,89]. Thus, Dab2 plays a key role in clathrin-mediated endocytosis by acting as a cargo specific adaptor protein which sorts and links membrane receptors to the endocytic machinery at the plasma membrane. Myosin VI co-localises with Dab2 in clathrin coated pits and vesicles in the apical domain of polarised cells suggesting that together they are involved in the early stages of clathrin mediated endocytosis such as receptor clustering and coated pit formation [48,45,49].

Optineurin; FIP-2; NRP (Nemo Related Protein) is a 67-kDa protein with predicted coiled coil regions, two leucine zipper domains and a putative Zn finger [90]. Optineurin binds to Huntingtin, the large protein involved in the neurodegenerative disorder, Huntington’s disease [91] and to Rab8, a small GTPase implicated in vesicular transport from the *trans*-Golgi network to the plasma membrane [55]. Optineurin and Rab8 link myosin VI to the Golgi complex and together they are responsible for maintaining the Golgi ribbon-like structure and for secretion from the Golgi to the plasma membrane [54].

GIPC (homologous to GLUT1 C-terminal-binding protein, SEMCAP-1 and synectin) binds to a number of transmembrane receptors such as the LDL receptor megalin [92], the human lutropin receptor (LHR) [93] and the β-1 adrenergic receptor [95] and it is likely that myosin VI and GIPC are involved in the endocytosis of these receptors [96]. The myosin VI-small or no insert isoforms co-localise with GIPC in uncoated endocytic vesicles and appear be involved in their movement through the cortical actin filament network below the plasma membrane [45,97].

SAP97 (postsynaptic density-95 protein) contains three PDZ domains (protein interaction domains), a SH3 domain (src homology 3) and a region with a guanylate kinase like sequence [98]. It binds to the Glu1 subunit of AMPA-type glutamate receptors in synapses and may have a role in the trafficking of AMPA receptors to the plasma membrane [99,100]. SAP97 interacts with myosin VI and immunoprecipitates as a complex from neuronal cells together with myosin VI and GluR1 [101]. Myosin VI may be involved in the clathrin-mediated endocytosis of AMPA receptors or in the exocytic transport or recyling of AMPA receptors to the cell surface and its absence in the Snell’s waltzer knock-out mouse leads to defects in synaptic structure and astrogliosis [102].

These binding partners all bind to two ‘hot spots’ either the RRL or WWY specific sites on the C-terminal cargo binding tail domain (Table 1) and may thus regulate the functions of myosin VI by helping to unfold and stabilise the tail and by recruiting the active molecule to specific intracellular locations [49]. The mutation of hydrophobic residues W to L in the Dab2 binding site (WWY to WLY) abolishes Dab2 binding to myosin VI indicating that the binding is relatively weak and probably transient. This binding affinity however maybe increased by binding to PIP_2_ [49] and other endocytic proteins. Low affinity binding motifs are present in endocytic proteins [103] and it has been suggested that low affinity protein-protein interactions are essential for the rapid and accurate coordination between the vast range of components involved in membrane trafficking pathways such as endocytosis [104,105].

### Inserts in the motor domain

Myosin VI has two unique inserts in its motor domain (head) not present in any other class of myosin, that regulate motor activity and directionality of movement along actin filaments. One insert (22 aa) near the switch 1 loop in the ATP binding region greatly decreases the ADP off rate and thus slows the rate of ATP rebinding [26]. It has been postulated that if myosin VI functions as a processive dimer then slower ATP rebinding would mean that the lead head would remain attached to actin for a longer time thus allowing mechanical coordination (gating) between the two heads to occur, which is essential for processive movement [23]. The second insert (53 aa) is an integral part of the converter region in myosin VI and contains a uniquely bound calmodulin [26,20,27]. It has recently been conclusively demonstrated that this insert is the sole determinant responsible for the reverse movement exhibited by myosin VI [106,107]. If this insert is missing then myosin VI moves like all the other myosins towards the plus end of actin filaments. Since this insert, called the reverse gear, is absent in all the other myosins expressed in mammalian cells, it means that no other myosin is able to move in the minus end direction on actin at least using such a reverse gear mechanism. So how is the Snell’s waltzer mouse, missing myosin VI, able to survive? What other motor proteins, mechanisms or pathways are able to compensate for the absence of myosin VI? Answers to these and many other intriguing questions may soon be forthcoming.

## Conclusions

Establishing precisely the mechanisms how myosin VI functions and is regulated in the many processes it is involved in different cells will help our understanding how it may operate in diseases such as cancer and deafness and will we hope aid in the development of intervention strategies and the identification of potential drug targets. The multiple functions of the unique retrograde motor myosin VI appear to be regulated in a number of ways; for example by the presence or absence of inserts in both the tail and motor domains, by PIP_2_/liposome binding, by phosphorylation, by calcium binding, by binding partners and possibly by other signals and modifications, which may together modulate its functions in the cell. In addition for specific functions such as anchoring or tethering myosin VI may operate as a monomer, whereas in trafficking pathways it may function as a dimer or as raft of monomers. Its assembly state may also be determined by the specific myosin VI isoform expressed, by the type of cell, e.g., polarised versus non-polarised, and by the nature and organisation of the cytoskeletal architecture in which it needs to function. Furthermore myosin VI does not operate in isolation and a host of other myosins, microtubule motors and specific binding partners are also likely to regulate its functions. Answers to these and many more basic questions need to be established before a full picture emerges of how myosin VI functions and is regulated in cells.

## Figures and Tables

**Table 1:** Myosin VI binding partners: their cellular locations, binding sites on myosin VI and proposed function

Binding partner	Cellular location	Binding site	Proposed function
Dab2	Plasma membrane, clathrin-coated pits and vesicles	WWY	Endocytosis of LDLR, tumour suppressor signalling pathways cell adhesion & movement
Optineurin	Cytoplasmic vesicles, Golgi complex	RRL	Exocytosis/secretion, Golgi morphology
GIPC	Endocytic vesicles, Golgi region	RRL	Endocytosis, receptor trafficking at Golgi, cell migration, cytokinesis
SAP97	Neuronal synaptic sites, cell adhesion sites	Unknown	Trafficking and endocytosis of AMPA receptors, cell-cell adhesion
T6BP/NDP52	Vesicles in perinuclear Golgi region, focal adhesions	RRL	Secretion, cell signalling, cell adhesions and ruffling
LMTK2	Cytoplasmic vesicles, Golgi region	WWY	Serine/threonine kinase endocytic recycling
